# Supplementary material for: Metabolomics-Based Profiling, Antioxidant Power, and Uropathogenic Bacterial Anti-Adhesion Activity of SP4TM, a Formulation with a High Content of Type-A Proanthocyanidins
Source: Antioxidants (Basel). 2022 Jun 23;11(7):1234. doi: 10.3390/antiox11071234 (PMC9312030; doi:10.3390/antiox11071234)
Supplement: Supplementary file 1 [file antioxidants-11-01234-s001.zip › antioxidants-1725302-supplementary.pdf]

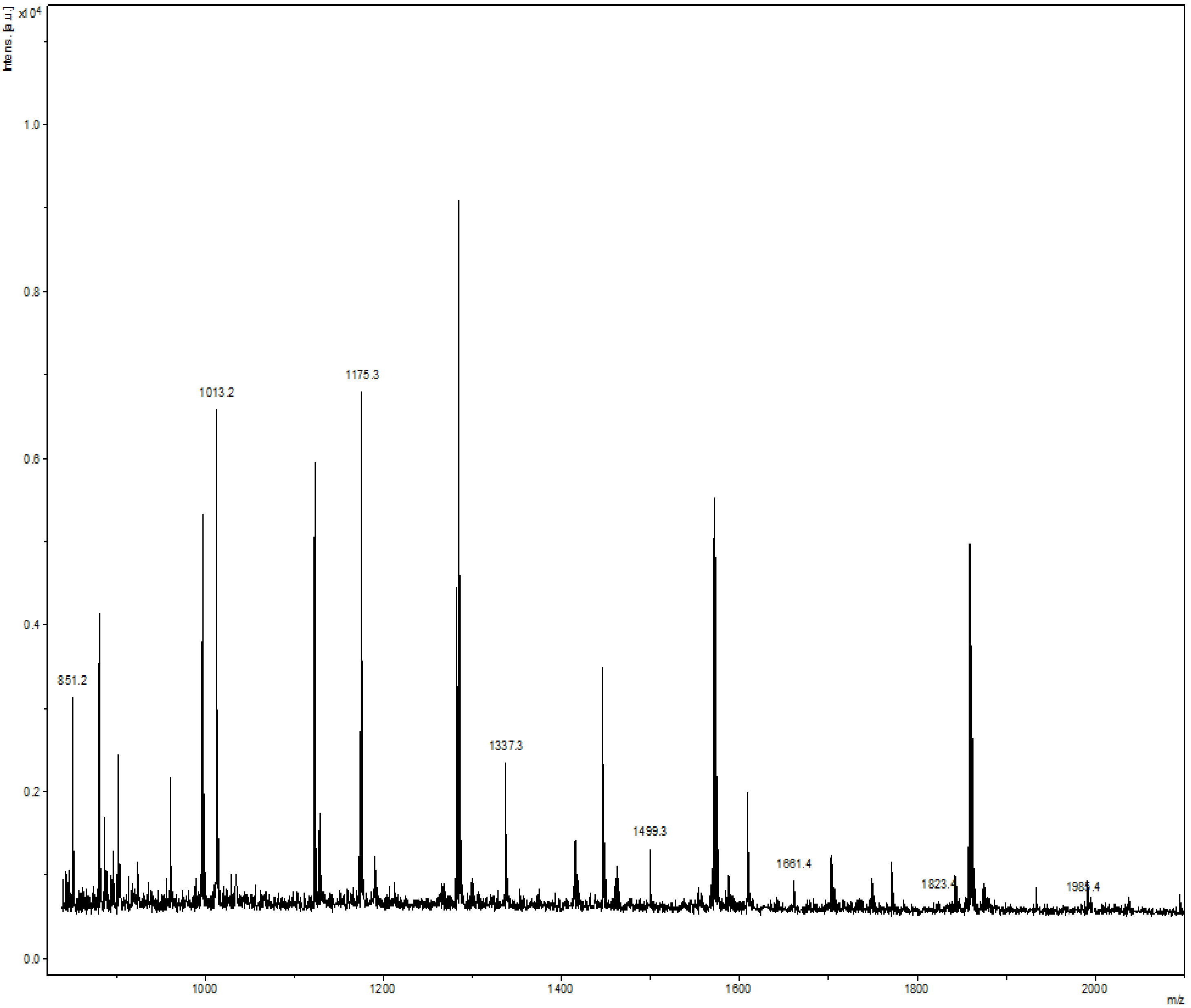

Figure S1. Positive reflectron mode MALDI-TOF MS oligosaccharide series of repeating hexoses from 5 degrees of polymerization (*m/z* 851) to 12 degrees of polymerization (*m/z* 1985). Masses are detected as sodium ion adducts $[M+Na]^+$.
